# Translation and validation of the French version of the Child Perceptions Questionnaire for children aged from 8 to 10 years old (CPQ _8-10_)

**DOI:** 10.1186/s12955-018-0907-x

**Published:** 2018-05-03

**Authors:** Marie-Laure Boy-Lefèvre, Noéline Razanamihaja, Sylvie Azogui-Lévy, Andréa Vigneron, Laurence Jordan, Ariane Berdal, Muriel de la Dure-Molla

**Affiliations:** 1 Dental Faculty of the University Paris Diderot, Paris, France; 2 Reference Center of Oral Rare Diseases O-Rares, Hopital Rotschild, Assistance Publique-Hopitaux de Paris, Paris, France; 3 Laboratory/Educations and Health Practices, EA 3412, University Paris 13, Villetaneuse, France; 4 CNRS UMR 8247-IRCP - Chimie ParisTech, Paris, France; 5 INSERM U 1138 –“Molecular Oral Physiopathology”, University Paris Diderot, Paris, France; 6 INSERM UMR-S1163- “Molecular and Physiopathological Bases of Osteochondrodysplasia”-Institut IMAGINE, Necker, University Paris Descartes, Paris, France

**Keywords:** Oral health quality of life, Children 8-10 years old, France, Reliability, Validity

## Abstract

**Background:**

The Child Perceptions Questionnaire (CPQ) belongs to a set of questionnaires measuring Child Oral Health Quality of Life (COHQOL). The CPQ is used to collect the perceptions of children on the impact of oral diseases on their quality of life. This cross-sectional study was aimed to translate the CPQ_8-10_ into French language and evaluate its psychometric properties.

**Methods:**

The translation process complied with international recommendations. The final French version was tested on children aged 8-10 years old attending consultations in a Parisian public hospital and divided into three groups: children with oral-facial clefts, children with dental anomalies linked to a rare disease other than clefts and children presumed to be healthy and without anomalies. The internal consistency relating to the reliability of CPQ_8-10_ was evaluated by Cronbach’s alpha. The intra-class correlation was used to measure reproducibility at the test-retest level. Construct validity was evaluated by Spearman’s correlation and tested using factor analysis. The discriminant validity was assessed using Kruskall Wallis test. Criterion validity was calculated using Spearman’s correlation.

**Results:**

One hundred seventy-six children participated in this study. During the translation process, minor changes were made. The French version showed good reliability with a Cronbach’s alpha of 0.81 for the total scale. The ICC of the test-retest was excellent (=0.90) demonstrating good reproducibility. The construct validity was acceptable with a statistically significant correlation between the scores of the French-CPQ_8-10_ and the evaluation of oral health (*r* = 0. 381 and *p* < 0.001) and its impact on oral health quality of life (*r* = 0.363 and *p* < 0.001). The loading weights obtained in the Exploratory Factor Analysis showed that this model revealed seven factors with eigenvalue greater than 1, explaining the 63,89% of the cumulative variance. The differences observed between the scores of the study groups revealed good discriminant validity. Criterion validity was supported by significant association between CPQ scores and pain.

**Conclusion:**

The French-CPQ_8-10_ is reliable and valid for use with the children of this age group.

**Electronic supplementary material:**

The online version of this article (10.1186/s12955-018-0907-x) contains supplementary material, which is available to authorized users.

## Background

The concept of oral health quality of life is raising increasing interest in the field of oral health care. Oral infections can have a negative impact on the quality of life of children undergoing full motor, intellectual and social development. Questionnaires for evaluating perceptions on the quality of life linked to children’s health were initially developed to be filled-in by parents and those responsible for children, considering that the latter were unable to evaluate abstract feelings. However, over the last few decades, studies have shown that children are able to express their feelings and that it is important to evaluate them. Questionnaires were therefore developed to evaluate quality of life perceived by children, linked to their state of oral health. In France, the use of oral health quality of life questionnaires, particularly for children, is still very limited despite them being very useful to evaluate the impact of oral pathologies, and treatment effect on the children quality of life and subsequently help policy making, promote oral health strategies and improve oral health care.

One of the first tools for measuring the oral health quality of life of children and one of the most frequently used is the series of COHQoL questionnaires, i.e. Child Oral Health Quality of Life Questionnaire. It is composed of three self-questionnaires: the Child Perceptions Questionnaires (CPQ) formulated as a function of children’s ages (CPQ _6-7_, CPQ_8-10_, CQP_11-14_) and also incorporates two questionnaires on the perception of the parents (P-CPQ) and the family impact scale (FIS). The series of COHQoL questionnaires was developed in English, in Canada [1, 2].

CPQ_8-10_ was firstly chosen among these age groups questionnaires; 8-10 years old children show a mixed denture and this age group is particularly interesting for cognitive properties*.*

The CPQ_8-10_ has already been translated and validated in several countries: China [3], Brazil [4], Denmark [5], Mexico [6], Bosnia and Herzegovina [7], and Korea [8]. It has been shown that this questionnaire has good psychometric characteristics in terms of reliability and validity for use with the children of this age group. CPQ_8-10_ has not yet been translated into French.

The aim of this study was to evaluate the reliability and validity of a version of the CPQ_8-10_ translated and cultural adapted in the French language for use on children in France.

## Methods

### The questionnaire

The original version of the CPQ_8-10_ has 25 items and is self-administered. The questionnaire begins by asking about the child’s age and gender. The 25 following items (Q1 to Q25) form the true core of the questionnaire and are divided into 4 domains: oral symptoms (OS) with 5 items, functional limitations (FL) with 5 items, emotional well-being (EWB) with 5 items, and social well-being (SWB) with 10 items. The questionnaire also contains two more global questions on the child’s perception of their oral health and on the way in which oral/oral facial conditions affect their general well-being. The items focus on the frequency of the events that have occurred during the 4 weeks before the questionnaire is administered.

The responses are expressed on a 5point Likert scale (ranging from 0 to 4). The respondents can choose between: “never”, score 0; “once or twice”, score 1; “sometimes”, score 2; “often”, score 3; and “every day or nearly every day”, score 4.

For the global question on the general perception of oral health, the responses had to be chosen between five scores: 0 = excellent, 1 = very good, 2 = good, 3 = average, 4 = poor. Regarding the second question: “how much does oral health affect your daily life?” the following measurement scale was given: 0 = not at all, 1 = very little, 2 = a little, 3 = a lot, 4 = very much.

The scores of the domains and items composing them were added to build the total score of CPQ_8-10_ for each child. The total score varied from 0 (no impact on the state of oral health quality of life) to 100 (maximum impact of state of oral health quality of life).

### Method

The original version of the CPQ_8-10_ was obtained using an article available with open access, shared by the terms of “Creative Commons License” which allowed its use without restriction, distribution and reproduction, with the necessary condition of citing the authors’ works.

#### Study population

This cross-sectional study was performed in the framework of the Odontology Centre of the Hôpital Rothschild in Paris (Assistance Publique-Hôpitaux de Paris). The study population gathered three groups of children: a group of children visiting the Paediatric Odontology department for consultation (group presumed healthy, without dental or oral-facial anomalies) and two groups of children referred to and attending their first consultation in the framework of the Rare Facial and Oral Cavity Malformations Reference Centre: an initial group of children with oral-facial clefts (lip and/or lip-palate), and a second group of children subject to dental anomalies linked to a rare disease other than oral-facial clefts (dental anomalies of number, shape and/or structure).

According to the recommendations of Everitt [9], we opted for a sample size ranging from 100 to 200.

To be included in the study the children had to be aged from 8 to 10 years old and know how to write and read, and speak French fluently.

### Adaptation and translation of the CPQ _8-10_

#### Translation-back translation

The questionnaire was translated according to the steps of the translation and back translation process, and to the transcultural adaptation recommended by Beaton et al. [10] and Guillemin et al. [11]. Indeed, Guillemin et al. [11] recommended having at least two independent translators. The translation from English to French was carried out by two translators, including a certified translator (linguist), and a dentist (for the medical terms), both are French mother tongue speakers bilingual and bicultural. The translations were performed independently. A committee composed of the translators and the investigators met to compare the versions translated into French and then proceeded to carry out a cultural adaptation of several of the terms giving rise to discussion and reaching consensus. At the end of this meeting, the first French version of the CPQ_8-10_ questionnaire was obtained.

Two new translators who were both totally unaware of the existence of the original version back translated this first version into English. Both versions, the original version and the back translated version, were then compared with each other by the evaluation committee and minor adjustments were made, justifying the quality of the translations carried out. Finally, a penultimate version was obtained (Additional files 1).

#### Pre-test procedure

The penultimate version was subjected to a pre-test on a group of 14 children aged 8 to 10 attending paediatric dentistry and who did not belong to the final sample, in order to discuss the pertinence of the items for this age-group and to identify problems of comprehension for the children. The observational monitoring pretesting procedure was applied. The investigators submitted the questionnaire to each child who responded individually to perform the face validity. In this way, facial expressions were noted on observation sheets and the children’s difficulties were identified orally and discussed with them afterwards. At the end of the face validity measure, the evaluation committee composed of the translators and dentists participating in the study and as experts, met again and formulated the final French version of the questionnaire (French-CPQ _8-10_), by taking into account several points that had led to questions from the children. A part from several minor adjustments, this version had the same number of items and domains as the other versions translated into other languages. Finally, the content validity (items and domains) of the French version was evaluated by the experts.

#### Statistical analysis

The data were processed and analysed using the SPSS software, version 24. Descriptive statistical analyses were performed for the mean values, standard deviations, the total scores and the scores of domains and items, the latter having been added to provide a total score for each child. Questionnaires with missing answers for more than 2 items will be excluded from further analysis. Item answers with missing values were recorded as 0 (zero).

### Reliability and validity assessment of the French version of the CPQ_8-10_

#### Reliability (Reproducibility)

Test-Retest and Test of internal consistency assessed the reliability of the French-CPQ_8-10_.

***Test-Retest***-: The French-CPQ _8-10_ was tested on 36 children and was subjected to a retest 2 weeks afterwards children who therefore completed the same questionnaire twice. Before the retest, investigators asked to the respondents if anything has changed about their oral health status. All respondents confirmed no change in their oral health status.

In this study, the Intra-Class Correlation (ICC) was used for the test-retest reliability of the questionnaire. An ICC, higher than 0.70, showed good reproducibility and was judged excellent if > 0.80 [12].

The ***internal consistency*** of the French-CPQ_8-10_ was evaluated by Cronbach’s alpha coefficient [13]. The value of Cronbach’s alpha ranged from 0 to 1.0. In most cases, this value should be ≥0.70 but a value between 0.60 and 0.70 was also considered acceptable [14, 15].

***Construct validity*** was analysed through convergent validity and discriminant validity. The Spearman’s correlation coefficient was used to test the convergent validity. Correlations were analysed between total scale and subscales scores with respectively oral health perceived status and overall well-being global indicators.

In order to evaluate the construct validity and assess whether the allocation of items in the domains corresponds to their distribution in the original questionnaire, an Exploratory Factor Analysis (EFA) using Varimax rotation was first conducted. A factor was considered important if its eigenvalue exceeded 1.0. EFA was followed by a partial confirmatory analysis (PCFA). For this, normed chi-square was used; it was the value produced by dividing chi-square values by the degree of freedom (df), and several different model indices of practical fit were evaluated including the Normed Fit Index (NFI), Comparative Fit Index (CFI), Tucker Lewis Index (TLI) [16], and Root Mean Squared Error of Approximation (RMSEA) [17]. A normed chi-square score < 3 was considered a good model [18]. An ideal score of CFI, NFI was >.70, TLI was >.90, and RMSEA scores <.10 were indicative of a good model [19].

The ***discriminant validity*** was evaluated using the non-parametric Kruskall Wallis test to determine whether the children with oral-facial clefts and/or presenting dental anomalies linked to a rare disease perceived more impact on their quality of life. The statistical significance of the results was fixed at *p* < 0.05.

***Criterion validity*** was calculated by comparing the correlations between CPQ scores and the variable pain obtained from the Q1, using Spearman’s correlation coefficient [20].

### Ethical considerations

This study was declared to the CNIL and obtained authorisation of the Research Project Ethics Evaluation Committee of the Hôpital Robert Debré (AP-HP), Paris (N° 2016/250-2.). An information sheet on the study was given to the children and to the parents of each child and the questionnaire was filled-in anonymously after obtaining agreement from the child and the written consent of their parents.

## Results

### Pretesting results

The translation/back translation step did not lead to any major difficulties. After the English-French translation was finished, the evaluation committee discussed six items so they could be better adapted to the French context. Furthermore, in Question 7: *“corn on the cob”* was changed to *“steak”,* since eating maize cobs is not usual for French children. The pre-test was performed on a group of 14 children. During the pre-test, in the question N°12, eight out of 14 children found difficult to understand the word “frustrated” relating to emotional well-being and it was replaced by “irrité”. Overall, the French version of the questionnaire did not lead to many questions from the children. The experts found the items and domains relevant to French culture (Additional file 1).

### Characteristics of the study population

In all, the study population was composed of 176 children aged from 8 to 10 years old attending consultations at the public hospital and whose parents had consented to their participation in the study. The children, aged 8, 9 and 10 years old, represented 33, 5%, 25, 6% and 40, 9%, respectively, with as many girls as boys (Table 1).

**Table 1:** Characteristics of children

	*N*	%
Age		
8 years old	59	33.5
9 years old	45	25.6
10 years old	72	40.9
Gender		
Female	88	50.0
Male	88	50.0
Total	176	100.0

All the 176 children answered the CPQ 8-10.

### Reliability of the French-CPQ_8-10_

The ICC of the test-retest was 0.91 for the total score with values between 0, 81 (EWB) and 0.87 (FL) for the domains. Cronbach’s alpha was 0.81 for the total scale and for the domains the values were 0.78 for Oral Symptoms and Functional Limitations, and 0.77 for Emotional Well-Being and 0.74 for Social Well-Being (Table 2).

**Table 2:** Descriptive statistics and reliability tests of French-CPQ_8-10_ (internal consistency of the total scale and the four subscales)

Variables	Items number	Mean (SD)	Cronbach’s alpha	ICC	95% IC
Total scale	25	14.1(12.8)	0.81	0.91	0.81-0.95
Subscales					
Symptômes oraux (Oral Symptoms/OS)	5	4.7(3.7)	0.78	0.81	0.73-0.87
Limitations fonctionnelles (Functional Limitations/FL)	5	2.8(3.5)	0.78	0.87	0.82-0.91
Bien-être émotionnel (Emotional Well-Being /EWB)	5	3.0(4.0)	0.77	0.81	0.73-0.87
Bien-être social (Social Well-Being/SWB)	10	3.6(4.8)	0.74	0.83	0.75-0.88

#### Construct validity

The correlation between the total score of the CPQ_8-10_, the self evaluation of the oral health and overall well-being are provided in Table 3 by positive and statistically significant Spearman correlation coefficients for the total scale (*r* = 0.38, *p* < 0.001; *r* = 0.36 p < 0.001 respectively). However, a lower correlation coefficient was obtained for the “Functional Limitations” domain (*r* = 0.14 with *p* > 0.05 and *r* = 0.17, *p* < 0.05 respectively).

**Table 3:** Construct validity –rank correlations between CPQ_8-10_ scores and global rating of Oral health and Overall well-being

	Global rating			
CPQ_8-10_	Oral health		Overall wellbeing	
	r*	*p*	r*	*p*
Total scale	0.38	< 0.001	0.36	< 0.001
Symptômes oraux (Oral Symptoms/OS)	0.23	0.002	0.28	< 0.001
Limitations fonctionnelles (Functional Limitations/FL)	0.14	< 0.001	0.17	0.028
Bien-être émotionnel (Emotional Well-Being /EWB)	0.28	< 0.001	0.27	< 0.001
Bien-être social (Social Well-Being/SWB)	0.47	< 0.001	0.47	< 0.001

r* Spearman’s correlation coefficient

Exploratory Factor Analysis was performed on the answers of the respondents to 25 items. The Kaiser-Meyer-Olkin test indicated good adjustment to latent factors (KMO = 0.837) and the Barlett’s Test of sphericity was significant at *p* < 0.001. These results confirmed that our data are suitable for Exploratory Factor Analysis (EFA).

Exploratory Factor Analysis (EFA) identified seven main factors which included 1-First factor: Feelings (items 11, 12, 13, 14, 15, 20, 24); 2-Second factor: Oral Functions (items 3, 4, 6, 7, 8); 3-Third factor: Oral Pain (items 1, 2, 10); 4-Fourth factor: Social relationship (items 17,19, 21, 22); 5-Fifth factor: Social Behaviour (items 18, 23); 6-Sixth factor: School Environment (items 9, 16, 25); 7-Seventh factor: Oral Health (item 5) and explained 63,89% of the common variance of QOL (Table 4).

**Table 4:** : Factor loadings of Child Perceptions Questionnaire _8-10_ in seven obtained factors

Questions	Components						
	Factor 1	Factor 2	Factor 3	Factor 4	Factor 5	Factor 6	Factor 7
	Feelings	Oral functions	Oral pain	Social relationship	Social behaviour	School environment	Oral health
1.Pain in teeth			.758				
2.Sores spots in mouth			.779				
3.Pain in teeth when drink/eat cold food		.606					
4. Food stuck in teeth		.523					
5.Bad breath							.785
6. Need longer time to eat		.687					
7.Hard time biting steaks		.473					
8.Trouble eating what would like to		.711					
9. Trouble saying words						.721	
10. Problem sleeping at night			.652				
11. Been upset	.770						
12. Felt frustrated	.713						
13. Been shy	.587						
14. Been concerned what people thing	.746						
15. Worried not good looking	.641						
16. Missed school						.527	
17. Hard time doing homework				.807			
18. Hard time paying attention					.415		
19. Not wanted to speak out loud				.540			
20. Tried not to smile/laugh	.541						
21. Not wanted to talk with others				.540			
22. Not wanted to be with others				.628			
23. Staying away from activities like sports..					.833		
24. Other children teased you	.613						
25. Other children ask you about your teeth						.607	

The Maximum Likehood Estimation (MLA) value, associated with the PCFA solution, was equal to 199.04 with 146 degree of freedom (df) and *p* value < 0.05, which was smaller than the corresponding null model khi^2^ of Bartlett ‘s test of sphericity of 1713.459 (*N* = 176; *p* < 0.05). Based on these two khi^2^ values, the normed khi^2^ value was 1.36 and the closed fit index values were calculated to be NFI = 0,883; CFI = 0,962; TLI = 0,923; and RMSEA = 0,045 (Additional file 2). The Visual Summaries of Fit of distributions of the residuals showed that the frequency distributions of the correlation residuals or covariance residuals had a normal shape.

#### Discriminant validity

Statistical differences (*p* < 0.001) were found between the total scores of the groups, the children with oral-facial clefts and those with rare dental anomalies (MR) had higher scores compared to those without any dental or oral/facial anomaly. The most marked differences concerned the “Social Well- Being” domain (Table 5).

**Table 5:** Discriminant validity: total scale scores and domains scores for children without rare disease condition and those with clefts and rare diseases other than clefts

CPQ_8-10_	PD*			CLEFTS			RD**			*P*
	Mean	Median	Standard deviation	Mean	Median	Standard deviation	Mean	Median	Standard deviation	
Total scale scores	11.83	8.00	11.08	17.73	13.00	11.21	20.21	13.5	16.73	< 0.001
Symptômes oraux (Oral Symptoms/OS)	4.63	4.00	3.91	4.41	3.50	2.92	5.38	4.00	3.92	0.538
Limitations fonctionnelles (Functional Limitations/FL)	2.35	1.00	3.35	3.95	3.00	3.56	3.65	2.00	4.18	0.048
Bien-être émotionnel (Emotional Well-Being /EWB)	2.51	1.00	3.51	2.95	1.50	3.99	4.82	2.50	5.18	0.012
Bien-être social (Social Well-Being/SWB)	2.34	1.00	3.38	6.41	6.00	4.61	6.41	6.00	4.61	< 0.001

PD* paediatric dentistry

CLEFTS

RD** rare diseases other than clefts

#### Criterion validity

The Table 6 showed the correlation between the scores of different subscales and variable “pain”, which was the sum of positive responses to the first question on the “*have you had pain in the mouth, teeth and jaw in the past month?*” as a result. There were positive correlation between the French-CPQ_8-10_ total scales score and the variable “pain” (*r*=. 55, *p* < 0.0001). Positive correlation were also found between all the domains of French-CPQ_8-10_ and the “pain” score.

**Table 6:** Criterion validity

CPQ8-10		PAIN	
		r*	*p*
	Total scale score	.55	.000
Subscales	Oral symptoms (OS)	.62	.000
	Functional limitations (FL)		
	Emotional well-being (EWB)	.39	.000
	Social well-being (SWB)	.38	.000

r* Spearman correlation coefficient

## Discussion

The objective of the study was to develop a French version of the Oral Health Quality of Life questionnaire (CPQ) for children aged from 8 to 10 years old. In our study, the whole translation/back-translation process led to the reformulation of certain sentences and the adoption of new terms such as “steaks” in the place of “corn on the cob” in view to adapting the expressions to the French cultural context without changing the meaning. Similar changes were also reported in other studies. Thus, in 2009, Wogelius et al., [5] for the validation of CPQ _8-10_ in Danish, and Barbosa et al. [4] in Brazilian, acknowledged having made adjustments for the translation of the CPQ8-10. The pre-test procedure was the same as that used by Aguilar-Diaz et al. in 2011 [6] for the Spanish version. It was also very similar to the method used by Jokovic et al. [2], during the development of the English version of the questionnaire, which incorporated a qualitative interview preceded the pre-test. This was done to note the children’ facial expressions of misunderstanding. A part from these minor adaptations, the entire contents of the questionnaire were judged applicable for use with native French speakers of the same age group. Furthermore, the acceptability of the questionnaire and its comprehension were demonstrated.

The number of item missing answers being very low, their effect was considered to be minor.

For the test-retest, the results showed good stability of the items’ responses. The ICC obtained for the four domains of this study were similar to those reported by Barbosa TS et al. [4]. The French-CPQ_8-10_, showed as well a good internal consistency with a high value of Cronbach alpha. These values were very similar to those found in the study of Wogelius et al. and Barbosa TS et al. [4, 5].

Spearman’s correlation showed a good constructed validity apart for the “Functional Limitations” domain for which a lower correlation was found in the present study. The original questionnaire validation and the Brazilian’s version reported as well non-significant correlation for FL domain [2, 4].

Based on the factor analysis of the French-CPQ_8-10_, 25 items in the scale were divided into seven factors, Thus, there were differences with the original version regarding item classification in domains: “Oral Symptoms” items were comprised in Factor 2 (Oral functions) and Factor 3 (0ral pain); “Social Well being” was divided into Factor 4 (Social relationship), Factor 5 (Social behaviour) and Factor 6 (School environment). “Functional Limitations” was mainly comprised in Factor 2 (Oral Functions) with 3/5 items. “Emotional Well being” domain with its 5 items was comprised in Factor 1 (Feelings). It might be assumed that these discrepancies could be related to linguistic and cultural specificities.

Following PCFA by using SPSS, the results indicated a validated model fit with all χ ^2^, *p* > 0.05, NFI > 0.8, CFI and TLI > 0.9 and RMSEA < 0.6 [19].

The discriminant validity expressed the apparent differences in the quality of life as a function of state of oral health. In this study, the clinical groups compared were children attending consultations for paediatric odontology, children presenting oral-facial clefts and a group subject to dental anomalies linked to a rare disease (dental agenesis, shape and structural anomalies). These groups were similar to those targeted by the study carried out in Denmark except for their healthy group, composed of schoolchildren [5]. In line with previous validations, the French-CPQ_8-10_ proved its capacity to distinguish groups of children with different states of oral health [2, 4–6].

Ideally, criterion validity would be measured with a gold standard when it is available but according to the literature [20], it is allowed to evaluate criterion validity by correlating the CPQ scores with external criteria, represented in this study by the sum of positive responses to the item investigating pain. Subjects with pain-associated condition presented higher impact on daily life as it was demonstrated by the discriminant validity. Positive correlation was found between all domains of French-CPQ8-10. The higher the correlation, the more valid is the measure for particular criteria.

The validated French-CPQ_8-10_ will be useful for clinicians to supplement information about the quality of dental care and for decision makers to improve dental services. This cross sectional study did not allow us to assess the responsiveness of questionnaire, which needs a longitudinal design study.

## Conclusion

The psychometric properties of the French version of the CPQ 8-10 were satisfactory. Its reliability and validity are such that it can be used with children aged from 8 to 10 years old in France. The validation of such an instrument presents a real advantage for performing future studies on the impact of dental health on the quality of life of these children in France.

Moreover, given the cross-sectional design of the study, the results concerned only descriptive, comparative and discriminative analysis. Therefore, further longitudinal research is required to explore the responsiveness of the French-CPQ _8-10_ (Additional file 2).

## Additional files

Additional file 1: Final french version of the (CPQ _8-10_) questionnaire. (DOC 73 kb)

Additional file 2: Partial Confirmatory Factor Analysis. (DOC 28 kb)

## References

[1] Jokovic A, Locker D, Stephens M, Kenny D, Tompson B (2002). Validity and reliability of a questionnaire for measuring child oral-health-related quality of life. J Dent Res.

[2] Jokovic A, Locker D, Tompson B, Guyatt G (2004). Questionnaire for measuring oral health-related quality of life in eight- to ten-year-old children. Pediatr Dent.

[3] McGrath C, Pang HN, Lo EC, King NM, Hagg U (2008). Translation and evaluation of a Chinese version of the child oral health-related quality of life measure. Int J Paediatr Dent.

[4] Barbosa TS, Tureli MC, Gaviao MB (2009). Validity and reliability of the child perceptions questionnaires applied in Brazilian children. BMC Oral Health..

[5] Wogelius P, Gjorup H, Haubek D, Lopez R, Poulsen S (2009). Development of Danish version of child oral-health-related quality of life questionnaires (CPQ8-10 and CPQ11-14). BMC Oral Health.

[6] Aguilar-Díaz FC, Irigoyen-Camacho ME (2011). Validation of the CPQ 8-10ESP in Mexican school children in urban areas. Med Oral Patol Oral Cir Bucal.

[7] Hadzipasic-Nazdrajic A (2012). Validation of the child perceptions questionnaire 8-10 in Bosnia and Herzegovina. Mater Sociomed.

[8] Shin HS, Han DH, Shin MS, Lee HJ, Kim MS, Kim HD (2015). Korean version of child perceptions questionnaire and dental caries among Korean children. PLoS One.

[9] Everitt BS (1975). Multivariate analysis: the need for data, and other problems. Brit J Psychiat.

[10] Beaton DE (2000). Guidelines for the process of cross-cultural adaptation of self-report measures. Spine.

[11] Guillemin F, Bombardier C, Beaton D (1993). Cross-cultural adaptation of health related quality of life measures: literature review and proposed guidelines. J Clin Epidemiol.

[12] Wilson-Genderson M, Broder HL, Philips C (2007). Concordance between caregiver and child reports of children’s oral health related quality of life. Community Dent Oral Epidemiol.

[13] Cronbach LJ (1951). Coefficient alpha and the internal structure of tests. Psychometrika.

[14] Bland JM, Altman DG (1997). Statistics notes: Cronbach’s alpha. BMJ.

[15] Nunnally JC (1978). Psychometric theory.

[16] Tucker LR, Lewis C (1973). A reliability coefficient for maximum likelihood factor analysis. Psychometrika.

[17] Bollen (1989). Structural equations with latent variables.

[18] Bentler PM, Bonett DG (1980). Significance tests and goodness-of-fit in the analysis of covariance structures. Psych Bull.

[19] Hu L, Bentler PM (1999). Cutoff criteria for fit indexes in covariance structure analysis : conventional criteria versus alternatives. Struct Equ Model.

[20] Nunnally JC, Bernstein IH (1994). Psychometric theory.

