# Supplementary material for: Translation and validation of the French version of the Child Perceptions Questionnaire for children aged from 8 to 10 years old (CPQ 8-10)
Source: Health Qual Life Outcomes. 2018 May 3;16:86. doi: 10.1186/s12955-018-0907-x (PMC5932841; doi:10.1186/s12955-018-0907-x)
Supplement: Supplementary file 1 — Final french version of the (CPQ 8-10) questionnaire. (DOC 73 kb) [file 12955_2018_907_MOESM1_ESM.doc]

| **Child Perceptions Questionnaire 8-10 (CPQ _8-10_) original**  version | **Questionnaire sur les perceptions de l’enfant âgé de 8 à 10 ans (CPQ _8-10_) Version française** |
| --- | --- |
| **Child oral health questionnaire**  Subject Number:  Hello,  Thanks for helping us with our study!  We are doing this study to understand better things that may happen to children because of their teeth and mouth.  Please Remember:  • Don’t write your name on the questionnaire.  • This is not a test and there are no right or wrong answers.  • Answer as honestly as you can.  • Don’t talk to anyone about the questions when you are answering them.  • No one you know will see your answers.  • Read each question carefully and think about the things that have happened to you in the past 4 weeks.  • Before you answer, ask yourself: “Does this happen to me because of my teeth or mouth?”  • Put an in the box beside the answer that is best for you.  DAY MONTH YEAR ____/____/____  **First, a few questions about you**  Today’s date:  Are you a boy or a girl?  **☐**Boy  **☐**Girl  How old are you? _________________  **Now a few questions about your teeth and mouth**  How often have you had:  1. Pain in your teeth or mouth in the past 4 weeks?  **☐**Never  **☐**Once or twice  **☐**Sometimes  **☐**Often  **☐**Everyday or almost every day  2. Sore spots in your mouth in the past 4 weeks?  **☐**Never  **☐**Once or twice  **☐**Sometimes  **☐**Often  **☐**Everyday or almost every day  3. Pain in your teeth when you drink cold drinks or eat foods in the past 4 weeks?  **☐**Never  **☐**Once or twice  **☐**Sometimes  **☐**Often  **☐**Everyday or almost every day  4. Food stuck in your teeth in the past 4 weeks?  **☐**Never  **☐**Once or twice  **☐**Sometimes  **☐**Often  **☐**Everyday or almost every day  5. Bad breath in the past 4 weeks?  **☐**Never  **☐**Once or twice  **☐**Sometimes  **☐**Often  **☐**Everyday or almost every day  In the past 4 weeks, how often have you:  6. Needed longer time than others to eat your meal because of your teeth or mouth?  **☐**Never  **☐**Once or twice  **☐**Sometimes  **☐**Often  **☐**Everyday or almost every day  7. Had a hard time biting or chewing food like apples, corn on the cob or steak because of your teeth or mouth?”  **☐**Never  **☐**Once or twice  **☐**Sometimes  **☐**Often  **☐**Everyday or almost every day  8. Had trouble eating foods you would like to eat because of your teeth or mouth?  **☐**Never  **☐**Once or twice  **☐**Sometimes  **☐**Often  **☐**Everyday or almost every day  9. Had trouble saying some words because of your teeth or mouth?  **☐**Never  **☐**Once or twice  **☐**Sometimes  **☐**Often  **☐**Everyday or almost every day  10. Had a problem sleeping at night because of your teeth or mouth?  **☐**Never  **☐**Once or twice  **☐**Sometimes  **☐**Often  **☐**Everyday or almost every day  **Some questions about your feelings**  In the past 4 weeks, how often have you:  11. Been upset because of your teeth or mouth?  **☐**Never  **☐**Once or twice  **☐**Sometimes  **☐**Often  **☐**Everyday or almost every day  12. Felt frustrated because of your teeth or mouth?  **☐**Never  **☐**Once or twice  **☐**Sometimes  **☐**Often  **☐**Everyday or almost every day  13. Been shy because of your teeth or mouth?  **☐**Never  **☐**Once or twice  **☐**Sometimes  **☐**Often  **☐**Everyday or almost every day  14. Been concerned what other people think about your teeth or mouth?  **☐**Never  **☐**Once or twice  **☐**Sometimes  **☐**Often  **☐**Everyday or almost every day  15. Worried that you are not as good-looking as others because of your teeth or mouth?  **☐**Never  **☐**Once or twice  **☐**Sometimes  **☐**Often  **☐**Everyday or almost every day  **QUESTIONS ABOUT YOUR SCHOOL**  In the past 4 weeks, how often have you:  16. Missed school because of your teeth or mouth?  **☐**Never  **☐**Once or twice  **☐**Sometimes  **☐**Often  **☐**Everyday or almost every day  17. Had a hard time doing your homework because of your teeth or mouth?  **☐**Never  **☐**Once or twice  **☐**Sometimes  **☐**Often  **☐**Everyday or almost every day  18. Had a hard time paying attention in school because of your teeth or mouth?  **☐**Never  **☐**Once or twice  **☐**Sometimes  **☐**Often  **☐**Everyday or almost every day  19. Not wanted to speak or read out loud in class because of your teeth or mouth?  **☐**Never  **☐**Once or twice  **☐**Sometimes  **☐**Often  **☐**Everyday or almost every day  **Questions about you being with other people**  In the past 4 weeks, how often have you:  20. Tried not to smile or laugh when with other children because of your teeth or mouth?  **☐**Never  **☐**Once or twice  **☐**Sometimes  **☐**Often  **☐**Everyday or almost every day  21. Not wanted to talk to other children because of your teeth or mouth?  **☐**Never  **☐**Once or twice  **☐**Sometimes  **☐**Often  **☐**Everyday or almost every day  22. Not wanted to be with other children because of your teeth or mouth?  **☐**Never  **☐**Once or twice  **☐**Sometimes  **☐**Often  **☐**Everyday or almost every day  23. Stayed away from activities like sports and clubs because of your teeth or mouth?  **☐**Never  **☐**Once or twice  **☐**Sometimes  **☐**Often  **☐**Everyday or almost every day  24. Other children teased you or called you names because of your teeth or mouth?  **☐**Never  **☐**Once or twice  **☐**Sometimes  **☐**Often  **☐**Everyday or almost every day  25. Other children asked you questions about your teeth or mouth?  **☐**Never  **☐**Once or twice  **☐**Sometimes  **☐**Often  **☐**Everyday or almost every day | **Questionnaire sur la santé bucco-dentaire de l’enfant**  *Numéro d’identification du sujet*  Bonjour,  Merci de nous aider en participant à cette étude!  Nous faisons cette étude pour mieux comprendre les problèmes que les enfants peuvent avoir avec leurs dents ou dans leur bouche.  *N’oublies pas :*  - il ne faut pas écrire ton nom sur le questionnaire  - ce n’est pas un contrôle donc il n’y a pas de bonne ni de mauvaise réponse  - de répondre aussi sincèrement que possible  - de ne pas discuter des réponses possibles avec quelqu’un d’autre lorsque tu réponds aux questions  - Personne parmi les gens que tu connais ne verra tes réponses  - Lis chaque question avec soin et penses à ce qui t’es arrivé au cours des 4 dernières semaines.  - Avant de répondre, demandes-toi « *Est ce que cela m’est bien arrivé à cause de mes dents ou de ma bouche ?*»  - Mets une croix dans la case devant la réponse qui te parait la meilleure pour toi  JOUR MOIS ANNEE ____/____/____  **Premièrement, quelques questions sur toi**  **E**s-tu un garçon ou une fille?  **☐**Garçon  **☐**Fille  Quel age as-tu ?……………  **Maintenant voici quelques questions au sujet de tes dents et de ta bouche**  Combien de fois as-tu eu :  1. *de douleur dans tes dents ou ta bouche au cours des 4 dernières semaines ?*  **☐**Jamais  **☐**Une ou deux fois  **☐**Parfois  **☐**Souvent  **☐**Tous les jours ou presque tous les jours  2. *des irritations dans ta bouche au cours des 4 dernières semaines?*  **☐**Jamais  **☐**Une ou deux fois  **☐**Parfois  **☐**Souvent  **☐**Tous les jours ou presque tous les jours  3. *de douleur dans les dents quand tu bois des boissons froides ou quand tu manges au cours des 4 dernières semaines?*  **☐**Jamais  **☐**Une ou deux fois  **☐**Parfois  **☐**Souvent  **☐**Tous les jours ou presque tous les jours  4. de la nourriture coincée dans tes dents au cours des 4 dernières semaines?  **☐**Jamais  **☐**Une ou deux fois  **☐**Parfois  **☐**Souvent  **☐**Tous les jours ou presque tous les jours  5. une mauvaise haleine au cours des 4 dernières semaines?  **☐**Jamais  **☐**Une ou deux fois  **☐**Parfois  **☐**Souvent  **☐**Tous les jours ou presque tous les jours  *Dans les 4 dernières semaines, combien de fois as-tu :*  6. eu besoin de plus de temps que les autres pour manger ton repas à cause de tes dents ou de ta bouche ?  **☐**Jamais  **☐**Une ou deux fois  **☐**Parfois  **☐**Souvent  **☐**Tous les jours ou presque tous les jours  7. eu des difficultés à mordre ou mâcher des aliments comme des pommes, du steak à cause de tes dents ou de ta bouche ?  **☐**Jamais  **☐**Une ou deux fois  **☐**Parfois  **☐**Souvent  **☐**Tous les jours ou presque tous les jours  8. eu des difficultés à manger des aliments que tu aurais voulu manger à cause de tes dents ou de ta bouche ?  **☐**Jamais  **☐**Une ou deux fois  **☐**Parfois  **☐**Souvent  **☐**Tous les jours ou presque tous les jours  9. eu des difficultés à prononcer certains mots à cause de tes dents ou de ta bouche ?  **☐**Jamais  **☐**Une ou deux fois  **☐**Parfois  **☐**Souvent  **☐**Tous les jours ou presque tous les jours  10. eu des difficultés à dormir la nuit à cause de tes dents ou de ta bouche ?  **☐**Jamais  **☐**Une ou deux fois  **☐**Parfois  **☐**Souvent  **☐**Tous les jours ou presque tous les jours  **Quelques questions au sujet de tes sentiments**  Dans les 4 dernières semaines, combien de fois **:**  11. As-tu été contrarié à cause de tes dents ou de ta bouche ?  **☐**Jamais  **☐**Une ou deux fois  **☐**Parfois  **☐**Souvent  **☐**Tous les jours ou presque tous les jours  12. T’es tu senti frustré à cause de tes dents ou de ta bouche ?  **☐**Jamais  **☐**Une ou deux fois  **☐**Parfois  **☐**Souvent  **☐**Tous les jours ou presque tous les jours  13. As-tu été timide à cause de tes dents ou de ta bouche ?  **☐**Jamais  **☐**Une ou deux fois  **☐**Parfois  **☐**Souvent  **☐**Tous les jours ou presque tous les jours  14. T’es tu senti préoccupé par ce que les autres personnes pensent de tes dents ou de ta bouche ?  **☐**Jamais  **☐**Une ou deux fois  **☐**Parfois  **☐**Souvent  **☐**Tous les jours ou presque tous les jours  15. As-tu été inquiet de ne pas être aussi beau que d’autres à cause de tes dents ou de ta bouche ?  **☐**Jamais  **☐**Une ou deux fois  **☐**Parfois  **☐**Souvent  **☐**Tous les jours ou presque tous les jours  **Questions au sujet de ton école**  Dans les 4 dernières semaines, combien de fois as-tu :  16. Manqué l’école à cause de tes dents ou de ta bouche ?  **☐**Jamais  **☐**Une ou deux fois  **☐**Parfois  **☐**Souvent  **☐**Tous les jours ou presque tous les jours  17. as-tu eu du mal à faire tes devoirs à cause de tes dents ou de ta bouche ?  **☐**Jamais  **☐**Une ou deux fois  **☐**Parfois  **☐**Souvent  **☐**Tous les jours ou presque tous les jours  18. as-tu eu du mal à te concentrer à l’école à cause de tes dents ou de ta bouche ?  **☐**Jamais  **☐**Une ou deux fois  **☐**Parfois  **☐**Souvent  **☐**Tous les jours ou presque tous les jours  19. est ce qu’il t’est arrivé de ne pas vouloir parler ou lire à haute voix en classe à cause de tes dents ou de ta bouche ?  **☐**Jamais  **☐**Une ou deux fois  **☐**Parfois  **☐**Souvent  **☐**Tous les jours ou presque tous les jours  **Questions sur ce que tu fais quand tu es avec d’autres personnes**  *Dans les 4 dernières semaines, combien de fois:*  20. as-tu  essayé de ne pas sourire ou rire quand tu es avec d’autres enfants à cause de tes dents ou de ta bouche ?  **☐**Jamais  **☐**Une ou deux fois  **☐**Parfois  **☐**Souvent  **☐**Tous les jours ou presque tous les jours  21. n’as-tu pas voulu parler avec d’autres enfants à cause de tes dents ou de ta bouche ?  **☐**Jamais  **☐**Une ou deux fois  **☐**Parfois  **☐**Souvent  **☐**Tous les jours ou presque tous les jours  22. n’as-tu pas voulu être avec d’autres enfants à cause de tes dents ou de ta bouche ?  **☐**Jamais  **☐**Une ou deux fois  **☐**Parfois  **☐**Souvent  **☐**Tous les jours ou presque tous les jours  23. as-tu évité les activités sportives et associatives à cause de tes dents ou de ta bouche ?  **☐**Jamais  **☐**Une ou deux fois  **☐**Parfois  **☐**Souvent  **☐**Tous les jours ou presque tous les jours  24. d’autres enfants se sont moqués de toi ou t’on insulté à cause de tes dents ou de ta bouche ?  **☐**Jamais  **☐**Une ou deux fois  **☐**Parfois  **☐**Souvent  **☐**Tous les jours ou presque tous les jours  25. d’autres enfants t’on posé des questions sur tes dents ou ta bouche ?  **☐**Jamais  **☐**Une ou deux fois  **☐**Parfois  **☐**Souvent  **☐**Tous les jours ou presque tous les jours |
