# Supplementary material for: Translation and validation of the French version of the Child Perceptions Questionnaire for children aged from 8 to 10 years old (CPQ 8-10)
Source: Health Qual Life Outcomes. 2018 May 3;16:86. doi: 10.1186/s12955-018-0907-x (PMC5932841; doi:10.1186/s12955-018-0907-x)
Supplement: Supplementary file 2 — Partial Confirmatory Factor Analysis. (DOC 28 kb) [file 12955_2018_907_MOESM2_ESM.doc]

**Partial Confirmatory Factor Analysis**

Indices of practical model fit

| Index | Khi^2^ | df | p | Khi^2^/df | NFI | **CFI** | **TLI** | **RMSE** | N |
| --- | --- | --- | --- | --- | --- | --- | --- | --- | --- |
| Value | 199.044 | 146 | .002 | 1.36 | .883 | .962 | .923 | .045 | 176 |
